# Vital Signs: Health Care–Associated Legionnaires’ Disease Surveillance Data from 20 States and a Large Metropolitan Area — United States, 2015

**DOI:** 10.15585/mmwr.mm6622e1

**Published:** 2017-06-09

**Authors:** Elizabeth A. Soda, Albert E. Barskey, Priti P. Shah, Stephanie Schrag, Cynthia G. Whitney, Matthew J. Arduino, Sujan C. Reddy, Jasen M. Kunz, Candis M. Hunter, Brian H. Raphael, Laura A. Cooley

**Affiliations:** ^1^Epidemic Intelligence Service, CDC; ^2^Divison of Bacterial Diseases, National Center of Immunization and Respiratory Diseases, CDC; ^3^Divison of Healthcare Quality and Promotion, National Center for Emerging and Zoonotic Infectious Diseases, CDC; ^4^Division of Emergency and Environmental Health Services, National Center for Environmental Health, CDC.

## Abstract

**Background:**

Legionnaires’ disease, a severe pneumonia, is typically acquired through inhalation of aerosolized water containing *Legionella* bacteria. *Legionella* can grow in the complex water systems of buildings, including health care facilities. Effective water management programs could prevent the growth of *Legionella* in building water systems.

**Methods:**

Using national surveillance data, Legionnaires’ disease cases were characterized from the 21 jurisdictions (20 U.S. states and one large metropolitan area) that reported exposure information for ≥90% of 2015 *Legionella* infections. An assessment of whether cases were health care–associated was completed; definite health care association was defined as hospitalization or long-term care facility residence for the entire 10 days preceding symptom onset, and possible association was defined as any exposure to a health care facility for a portion of the 10 days preceding symptom onset. All other Legionnaires’ disease cases were considered unrelated to health care.

**Results:**

A total of 2,809 confirmed Legionnaires’ disease cases were reported from the 21 jurisdictions, including 85 (3%) definite and 468 (17%) possible health care–associated cases. Among the 21 jurisdictions, 16 (76%) reported 1–21 definite health care–associated cases per jurisdiction. Among definite health care–associated cases, the majority (75, 88%) occurred in persons aged ≥60 years, and exposures occurred at 72 facilities (15 hospitals and 57 long-term care facilities). The case fatality rate was 25% for definite and 10% for possible health care–associated Legionnaires’ disease.

**Conclusions and Implications for Public Health Practice:**

Exposure to *Legionella* from health care facility water systems can result in Legionnaires’ disease. The high case fatality rate of health care–associated Legionnaires’ disease highlights the importance of case prevention and response activities, including implementation of effective water management programs and timely case identification.

## Introduction

*Legionella* is a waterborne bacterium responsible for Legionnaires’ disease, a severe pneumonia that occurs most frequently in susceptible persons, including those aged ≥50 years, former or current smokers, and those with chronic diseases or immunosuppression (*1*). Whereas approximately 9% of Legionnaires’ disease cases are fatal (*1*), mortality associated with health care–associated Legionnaires’ disease is higher, with reported case fatality rates (CFRs) historically as high as 46% (*2*). *Legionella* grows well in building water systems* that are not adequately managed, especially those where disinfectant levels are low, water is stagnant, or water temperatures are optimal for growth^†^ (*3*). Illness with Legionnaires’ disease most commonly occurs after inhalation of *Legionella*-containing aerosols from showerheads, certain medical equipment (e.g., respiratory equipment), cooling towers, hot tubs, hydrotherapy equipment, or decorative fountains (*4*). Less commonly, disease occurs from aspiration of *Legionella*-containing water (*5*). Only one case of probable person-to-person transmission has been reported (*6*).

The size and complexity of health care facility water systems and the vulnerability of the patient populations served by these facilities increase the risk for *Legionella* transmission and severe outcomes. A review of 27 Legionnaires’ disease outbreaks investigated by CDC during 2000–2014 indicated that health care–associated Legionnaires’ disease accounted for 33% of the outbreaks, 57% of outbreak-associated cases, and 85% of outbreak-associated deaths (*7*). In addition, 85% of all Legionnaires’ disease outbreaks were attributed to water system exposures that could have been prevented by effective water management programs.

Implementation of water management programs that prevent conditions conducive to *Legionella* growth and transmission, combined with rapid case identification and investigation, could prevent health care–associated Legionnaires’ disease cases and outbreaks (*8*–*10*). Health care facilities are ideally positioned to establish and maintain prevention and response activities because they can build upon existing infection control and patient safety activities.

Legionnaires’ disease cases are reportable to CDC. Fifty states, two large U.S. metropolitan areas, and five territories report basic demographic information to the National Notifiable Diseases Surveillance System (NNDSS) for all cases of legionellosis, which comprises two distinct clinical presentations: Pontiac fever, a mild influenza-like illness, and Legionnaires’ disease. NNDSS does not distinguish between the two presentations. In 2015, 6,079 cases of legionellosis were reported to NNDSS, although this number might be an underestimate because of underdiagnosis. The Supplemental Legionnaires’ Disease Surveillance System (SLDSS) receives more epidemiologic information, such as exposure to health care facilities, and does distinguish Legionnaires’ disease from Pontiac fever, but reporting to SLDSS is less complete.

The proportion of the U.S. Legionnaires’ disease cases associated with health care facilities has not been established. The objective of this analysis was to describe reported U.S. cases of health care–associated Legionnaires’ disease using surveillance data from 21 jurisdictions in 2015 to highlight the importance of Legionnaires’ disease prevention and response in health care facilities.

## Methods

The 20 states and one large metropolitan area^§^ that reported ≥90% of confirmed NNDSS legionellosis cases to SLDSS in 2015 were included in this analysis. Only confirmed Legionnaires’ disease cases from SLDSS, defined by the Council of State and Territorial Epidemiologists as laboratory confirmation of *Legionella* in a person with clinical illness compatible with Legionnaires’ disease (*11*), were analyzed.

Reported case exposures were categorized as health care–associated or not health care–associated. Cases were considered health care–associated if they occurred in a person who visited, worked, or stayed in a health care facility for any amount of time in the 10 days preceding symptom onset. Health care–associated Legionnaires’ disease cases were further classified as definite (continuous exposure to a hospital or long-term care facility for the entire 10 days preceding symptom onset) or possible (any exposure to a health care facility for a portion of the 10 days preceding symptom onset). Health care–specific exposure settings included hospitals, long-term care facilities (facilities providing a skilled need such as intravenous medication administration), clinics, and others (e.g., outpatient laboratories). Descriptive statistics were generated, and results are reflective of cases reported to SLDSS as of April 14, 2017.

## Results

Among 6,079 confirmed legionellosis cases reported to NNDSS, SLDSS received reports of 3,516 (58%), including 3,459 Legionnaires’ disease cases (Figure). Among the 3,459 Legionnaires’ disease cases, 2,809 (81%) were reported by the 21 jurisdictions included in this analysis, including 553 (20%) that were health care–associated.

**FIGURE:**
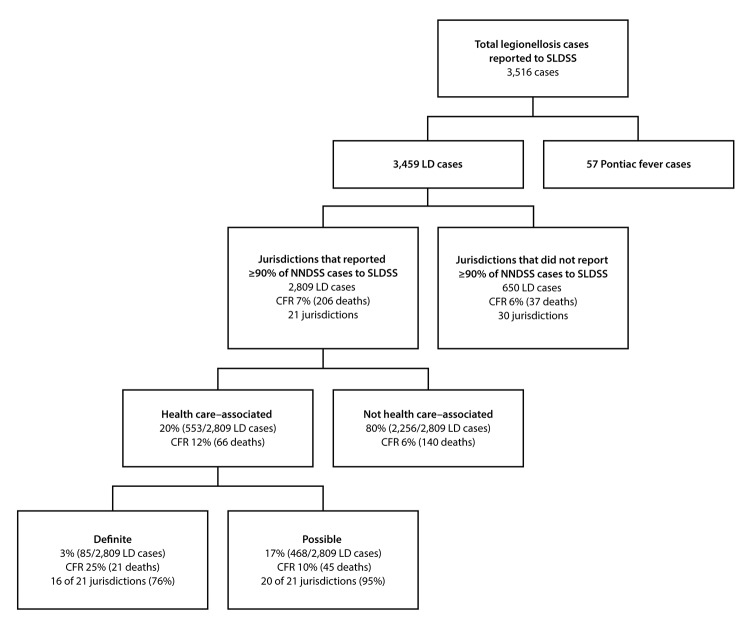
Categorization of confirmed cases of legionellosis*^,†^ reported to the Supplemental Legionnaires’ Disease Surveillance System, 2015 **Abbreviations:** CFR = case fatality rate; LD = Legionnaires’ disease; NNDSS = National Notifiable Diseases Surveillance System; SLDSS = Supplemental Legionnaires’ Disease Surveillance System. * Legionellosis cases include Legionnaires’ disease and Pontiac fever, a mild influenza-like illness. ^†^ A total of 6,079 cases of legionellosis were reported to NNDSS in 2015.

Among the 21 jurisdictions, 16 (76%) reported definite health care–associated cases (1–21 cases per jurisdiction); four of the remaining five reported possible health care–associated Legionnaires’ disease cases. Definite and possible health care–associated cases accounted for 3% and 17%, respectively, of all cases reported by the 21 jurisdictions (Figure). CFR was 12% overall for health care–associated Legionnaires’ disease cases (25% for definite and 10% for possible cases).

Among the 85 definite health care–associated Legionnaires’ disease cases, 68 (80%) were associated with long-term care facilities, 15 (18%) with hospitals, and two (2%) with both (Table 1). Definite health care–associated Legionnaires’ disease cases were reported in 72 facilities, including 15 hospitals and 57 long-term care facilities, and included one to six cases per facility. The majority of definite cases occurred in persons aged ≥60 years (75, 88%) (Table 2).

**TABLE 1:** Confirmed health care–associated Legionnaires’ disease,* by setting and likelihood that exposure to *Legionella* was from a health care facility water system — 21 U.S. public health jurisdictions,^†^ 2015

Type of facility	No. cases (%)		
	Definite^§^	Possible^¶^	Total
Hospital	15 (18)	227 (49)	**242 (44)**
Long-term–care	68 (80)	61 (13)	**129 (23)**
Clinic	0 (0)	123 (26)	**123 (22)**
Multiple**	2 (2)	44 (9)	**46 (8)**
Other^††^	0 (0)	13 (3)	**13 (2)**
**Total**	**85 (100)**	**468 (100)**	**553 (100)**

* Health care–associated Legionnaires’ disease includes both definite and possible cases in persons who visited, worked, or stayed in a health care setting for any amount of time in the 10 days preceding symptom onset.

^†^ Twenty-one jurisdictions that reported ≥ 90% of confirmed National Notifiable Diseases Surveillance System legionellosis cases to the Supplemental Legionnaires’ Disease Surveillance System in 2015: Alabama, Colorado, Connecticut, Georgia, Hawaii, Iowa, Kentucky, Maine, Michigan, Minnesota, Missouri, New Hampshire, New Mexico, New York, New York City, North Dakota, Ohio, Rhode Island, South Carolina, Texas, and Virginia.

^§^ Definite case of health care–associated Legionnaires’ disease was defined as laboratory-confirmed legionellosis in a patient with continuous exposure to a hospital or long-term–care facility for the entire 10 days preceding symptom onset.

^¶^ Possible case of health care–associated Legionnaires’ disease was defined as laboratory-confirmed legionellosis in a patient with any exposure to a health care facility for a portion of the 10 days preceding symptom onset.

** Multiple indicates two or more of the listed setting categories.

^††^ Other setting includes locations such as outpatient laboratories.

**TABLE 2:** Demographic characteristics of patients with confirmed Legionnaires’ disease*— 21 U.S. public health jurisdictions,^†^ 2015

Characteristic	No. cases (%)			
	Definite health care–associated (n = 85)		Possible health care–associated (n = 468)	Not health care–associated (n = 2,256)
**Age group (yrs)**				
0–29	0 (0)		16 (3.4)	59 (2.6)
30–39	1 (1.2)		10 (2.1)	148 (6.6)
40–49	2 (2.4)		35 (7.5)	322 (14.3)
50–59	7 (8.2)		111 (23.7)	596 (26.4)
60–69	18 (21.2)		125 (26.7)	557 (24.7)
70–79	23 (27.1)		88 (18.8)	321 (14.2)
80–89	18 (21.2)		67 (14.3)	197 (8.7)
≥90	16 (18.8)		15 (3.2)	53 (2.4)
Unknown	0 (0)		1 (0.2)	3 (0.1)
**Sex**				
Male	40 (47.1)		263 (56.2)	1,419 (62.9)
Female	45 (52.9)		200 (42.7)	820 (36.4)
Unknown	0 (0)		5 (1.1)	17 (0.8)
**Race**				
Black or African American	16 (18.8)		91 (19.4)	598 (26.5)
White	53 (62.3)		315 (67.3)	1,373 (60.9)
Asian	0 (0 )		5 (1.1)	20 (0.9)
American Indian/Alaska Native	0 (0)		2 (0.4)	12 (0.5)
Native Hawaiian/Pacific Islander	0(0)		1(0.2)	3(0.1)
Multiple	1 (1.2)		0 (0)	0 (0)
Unknown	15 (17.7)		54 (11.5)	250 (11.1)
**Ethnicity**				
Hispanic	3 (3.5)		29 (6.2)	159 (7.1)
Non-Hispanic	65 (76.5)		338 (72.2)	1,673 (74.2)
Unknown	17 (20.0)		101 (21.6)	424 (18.8)

* Definite health care–associated Legionnaires’ disease was defined as laboratory-confirmed legionellosis in a patient with continuous exposure to a hospital or long-term care facility for the entire 10 days preceding symptom onset. Possible health care–associated Legionnaires’ disease was defined as laboratory-confirmed legionellosis in a patient with any exposure to a health care facility for a portion of the 10 days preceding symptom onset. All other cases were considered not health care–associated.

^†^ Twenty-one jurisdictions that reported ≥90% of confirmed National Notifiable Diseases Surveillance System legionellosis cases to the Supplemental Legionnaires’ Disease Surveillance System in 2015: Alabama, Colorado, Connecticut, Georgia, Hawaii, Iowa, Kentucky, Maine, Michigan, Minnesota, Missouri, New Hampshire, New Mexico, New York, New York City, North Dakota, Ohio, Rhode Island, South Carolina, Texas, and Virginia.

Among 468 possible health care–associated Legionnaires’ disease cases, 61 (13%) were possibly associated with long-term care facilities, 227 (49%) with hospitals, 123 (26%) with clinics, 13 (3%) with other settings such as outpatient laboratories, and 44 (9%) with more than one setting. Possible health care–associated Legionnaires’ disease cases occurred in approximately 415 health care facilities and included one to 31 cases per facility.

## Conclusions and Comments

Although health care–associated Legionnaires’ disease is less common than some other health care–acquired infections, its impact on patients and affected health care facilities is considerable. For patients, health care–associated Legionnaires’ disease can result in high morbidity, mortality, and financial cost (*1*,*12*). For health care facilities, Legionnaires’ disease cases and outbreaks can involve substantial expense related to investigation, remediation, legal action, and reputational costs (*13*,*14*). Furthermore, compared with more common health care–acquired infections, general understanding of the necessary prevention and response measures for waterborne pathogens, such as *Legionella*, might be lacking.

In this analysis, definite health care–associated Legionnaires’ disease cases were reported by the majority of the 21 jurisdictions and occurred in 72 institutions. Although only 3% of reported Legionnaires’ disease cases from the 21 jurisdictions were definitely health care–associated, the CFR among these cases was high. Furthermore, the number of definite cases and facilities reported here is likely an underestimate of the actual case number, because some possible cases likely acquired their infection from a health care facility, and some infections were likely undiagnosed because of a lack of *Legionella*-specific testing. A larger number of definite cases were associated with long-term care facilities than with hospitals. One explanation for this might be that hospital stays are typically shorter (*15*) than the 10-day period used in this analysis to define a definite health care–associated case. Pending further research, other conclusions cannot accurately be drawn, and thus these findings should not be used to establish the level of risk among facility types.

In health care facilities, prevention of the first case of Legionnaires’ disease is the ultimate goal. This goal is likely best achieved by establishing and maintaining an effective water management program (*8*,*10*). In 2015, ASHRAE^¶^ issued guidance on water management programs (*3*). CDC and partners adapted this standard into a simpler format (https://www.cdc.gov/legionella/WMPtoolkit) that guides users such as health care facility leaders** or other decision makers through the steps needed for such a program. Most recently, the Centers for Medicare & Medicaid Services released a survey and certification memo stating that health care facilities should develop and adhere to ASHRAE–compliant water management programs to reduce the risk for *Legionella* and other pathogens in their water systems (*16*).

In general, the principles of effective water management include maintaining water temperatures outside the ideal range for *Legionella* growth, preventing water stagnation, ensuring adequate disinfection, and maintaining equipment to prevent scale, corrosion, and biofilm growth, which provide a habitat and nutrients for *Legionella* (*3*). Once established, water management programs require regular monitoring of key areas in the system for potentially hazardous conditions, and the use of prespecified responses to remediate such conditions if they are detected. The additional benefit of water management programs include the control of other water-related health care–associated infections such as those caused by nontuberculous mycobacteria. Programs need to be monitored for their efficacy in reducing risk across microbial species (*17*). Such ongoing monitoring is especially relevant because specific mitigation strategies, or partially implemented mitigation strategies, might control one pathogen at the expense of selecting for another (*18*).

Health care providers play a critical role in prevention and response by rapidly identifying and reporting cases. Legionnaires’ disease is clinically indistinguishable from other causes of pneumonia; a failure to diagnose a health care–associated case could result in a missed opportunity to prevent subsequent cases. *Legionella* should be considered as a cause of health care–associated pneumonia, especially for groups at increased risk, when other facility-related cases have been identified, or when changes in water parameters might lead to increased risk for Legionnaires’ disease. The preferred diagnostic procedure for Legionnaires’ disease is to concurrently obtain a lower respiratory sputum sample for culture on selective media and a *Legionella* urinary antigen test. Sputum should ideally be obtained before antibiotic administration and should not be rejected on the basis of specimen quality (e.g., lack of polymorphonuclear leukocytes or contamination with other bacteria), as sputa produced by patients with Legionnaires’ disease might not be purulent and contaminating bacteria will not negatively affect isolation of *Legionella* on selective media (*19*,*20*). The urinary antigen test only detects *Legionella pneumophila* serogroup 1, the most common cause of Legionnaires’ disease (*21*). Particularly in health care settings, cases of Legionnaires’ disease caused by other species and serogroups can occur. An isolate from culture is needed for the identification of these species and serogroups, as well as for molecular comparison of clinical to environmental isolates as part of investigations.

In addition to being critical partners in national Legionnaires’ disease reporting, public health jurisdictions have an influential role in prevention and response activities. Some public health departments or agencies might serve as a resource to facilities during the development, implementation, and evaluation of a water management program. Public health officials also play an important role in response, including outbreak identification, environmental assessment to determine *Legionella* exposure sources, and development of recommendations to prevent ongoing transmission. Hence, prompt reporting of Legionnaires’ disease cases to public health can facilitate a timely and effective response.

The findings in this report are subject to at least three limitations. First, data from more jurisdictions and more years would improve the accuracy of U.S. health care–associated Legionnaires’ disease case estimates. Second, the completeness of the health care exposure information in this data set was not assessed. For example, whether a substantial number of health care exposures were not reported or inaccurately reported is unknown. Finally, CFRs reported here might be biased by lack of information on Legionnaires’ disease deaths that occurred after reporting to CDC (resulting in CFR underestimation) or deaths of Legionnaires’ disease patients from other causes (resulting in CFR overestimation).

This report demonstrates that Legionnaires’ disease continues to result from exposures to health care facility water systems. The high case fatality rate of health care–associated Legionnaires’ disease underscores the need for effective prevention and response programs. Implementation and maintenance of water management programs, combined with rapid case identification and investigation, could reduce the number of health care–associated Legionnaires’ disease cases.

**Key Points**

• Legionnaires’ disease is a severe lung infection caused by breathing in small droplets of water that contain *Legionella* bacteria. Persons aged ≥50 years, current or former smokers, and those with chronic diseases or a weakened immune system are at higher risk for Legionnaires’ disease.

• *Legionella* grows well in building water systems that are not adequately managed such as those in which disinfectant levels are low or water temperatures are warm. Effective water management programs are recommended to prevent *Legionella* growth in buildings with large or complex water systems, including health care facilities.

• The size and complexity of health care facility water systems might increase the risk for *Legionella* growth. Such health care facilities also provide care to persons who might be more susceptible to Legionnaires’ disease because of their underlying risk factors.

• Legionnaires’ disease continues to occur in U.S. health care facilities. Sixteen of the 21 U.S. jurisdictions, including 72 health care facilities in this analysis, reported definite health care–associated cases of Legionnaires’ disease.

• One fourth of persons with definite health care–associated Legionnaires’ disease die.

• Prevention and response requires coordination among health care facility leaders, health care providers, and public health professionals. Instituting and maintaining effective water management programs are the principal prevention measures. Rapid patient identification with appropriate laboratory testing and prompt intervention might prevent additional cases from occurring.

• Additional information is available at https://www.cdc.gov/vitalsigns.

## Footnotes

* Large or complex water systems, where *Legionella* has more opportunity to grow and spread, are most often found in commercial, institutional, multiunit residential, health care, and industrial buildings, often with multiple stories. ASHRAE Standard 188 recommends water management for these types of buildings in addition to those in which vulnerable populations, such as immunocompromised or elderly persons, live or are treated.

† The temperature range most favorable for growth of *Legionella* is 77°F–108°F (25°C–42°C), although *Legionella* has been recovered from water with temperatures outside this range. For health care facilities, ASHRAE Guideline 12-2000 recommends storing and distributing cold water at temperatures <68°F (<20°C), whereas hot water should be stored at >140°F (>60°C) and circulated with a minimum return temperature of 124°F (51°C). In other settings, hot water should be stored at ≥120°F (≥49°C).

§ The 21 jurisdictions are Alabama, Colorado, Connecticut, Georgia, Hawaii, Iowa, Kentucky, Maine, Michigan, Minnesota, Missouri, New Hampshire, New Mexico, New York, New York City, North Dakota, Ohio, Rhode Island, South Carolina, Texas, and Virginia.

¶ Formerly known as the American Society of Heating, Refrigerating, and Air-Conditioning Engineers.

** Persons in leadership roles for prevention activities might include infection control practitioners, facility managers, hospital administrators, quality assurance staff members, or others who are ultimately responsible for implementing an effective water management program and for ensuring that ongoing communication regarding *Legionella* occurs between environmental health personnel, clinical staff members, and public health officials.
